# Trust and cooperative behavior: Evidence from the realm of data-sharing

**DOI:** 10.1371/journal.pone.0220115

**Published:** 2019-08-21

**Authors:** Paul C. Bauer, Florian Keusch, Frauke Kreuter

**Affiliations:** 1 Mannheim Centre for European Social Research (MZES), University of Mannheim, Mannheim, Germany; 2 Department of Sociology, University of Mannheim, Mannheim, Germany; 3 Joint Program in Survey Methodology, University of Maryland, College Park, Maryland, United States of America; 4 Institute for Employment Research, Nuremberg, Germany; Middlesex University, UNITED KINGDOM

## Abstract

Trust is praised by many social scientists as the foundation of functioning social systems owing to its assumed connection to cooperative behavior. The existence of such a link is still subject to debate. In the present study, we first highlight important conceptual issues within this debate. Second, we examine previous evidence, highlighting several issues. Third, we present findings from an original experiment, in which we tried to identify a “real” situation that allowed us to measure both trust and cooperation. People’s expectations and behavior when they decide to share (or not) their data represents such a situation, and we make use of corresponding data. We found that there is no relationship between trust and cooperation. This non-relationship may be rationalized in different ways which, in turn, provides important lessons for the study of the trust—behavior nexus beyond the particular situation we study empirically.

## Introduction

*Is trust related to cooperative behavior?* Social scientists have classically studied trust, conceptualized as a mental state and measured as such, because they assume that high levels of trust reflect a social reality in which people are more trustworthy and tend to cooperate more frequently [1, 2]. The idea that trust is linked to cooperation dates back to the earliest works on the concept [3, 4]. Only actors who trust each other should cooperate with each other, i.e., exchange information, resources, etc. Entering a cooperative relationship normally requires a certain level of trust, and the same is necessary to sustain that relationship. Sometimes trust scholars explicitly refer to the cooperation argument to defend their research, sometimes they implicitly assume it [5, 6]. However, to this day scholars debate whether such a link actually exists [7]. Our study is motivated by this debate and our contribution is three-fold.

First, we review past contributions to this debate as well as evidence that links trust to cooperation [7, 8]. Most of the respective findings stem from laboratory experiments, since here cooperation can be observed in a controlled environment. However, even among scholars pursuing lab experiments, debates ensued on how such lab experiments would need to be constructed so that the observed behavior could be regarded as cooperative behavior that is caused by trust. We show that current evidence can be interpreted much more meaningfully after having clarified certain conceptual issues. For instance, it makes a difference whether we measure “generalized trust” or trust, i.e., what can also be called situational or specific trust [9–11].

Second, we critically review this evidence and contrast it with an ideal experiment, i.e., a design one would choose to study the relationship between trust and cooperative behavior if there weren’t any practical or ethical constraints. This approach emphasizes various aspects that were either not highlighted at all or at least underemphasized in previous research. Importantly, it produces some new arguments on why previous evidence regarding this question is so mixed and suggests that the ideal—even if not attainable—may still provide a useful benchmark.

Third, after providing an overview of the limitations that characterize current evidence connecting trust and cooperative behavior, we present findings from a survey in Germany. For our study we identified a “real situation” that would allow us to observe both trust and cooperative behavior. A corresponding situation is when people share, i.e., entrust their data to others. In studying this situation we also contribute to a growing body of literature that investigates attitudes towards data sharing and data sharing behavior in modern digital societies [12–14].

Section 2 provides conceptual clarifications that are helpful in the discussion of trust and cooperation and presents an ideal setting of how our question would be studied without constraints. Section 3 summarizes and evaluates evidence on our research question that has been collected so far. Section 4 presents our methodological approach. Section 5 presents the results, which are summarized and discussed in the conclusion in Section 6.

## Theory, hypothesis and ideal experiment

There is relatively wide interdisciplinary agreement that trust describes a psychological state [15]. More specifically, trust can be defined as a *truster A’s (probabilistic) expectation that a trustee B will display behavior X in situation/context Y* [5, 16–18]. The fact that A prefers behavior X over some other behavior differentiates trust from a mere expectation. Thus, in contrast to *generalized trust* aka *social trust* aka *cross-situational trust* [17]—a generalized, situation-independent expectation—*trust* describes expectations that are tailored to situations. To clarify that *trust* and *generalized trust* are two different concepts, scholars often attach the qualification *situational* or *specific* to the former [17].

Cooperation, in turn, describes situations in which an individual willingly acts in a manner that contributes to the others’ welfare [19, 20]. In other words, a truster engages in cooperative behavior when she acts in a way that benefits the trustee or both. The cooperative behavior studied in the experimental literature is usually the truster’s behavior in the trust game [8, 21]. To make a clear conceptual distinction between trust and behavior, scholars sometimes call the latter trusting behavior. At the same time trusting behavior was rarely defined in relation to trust. For instance, it could be defined as behavior motivated by a particular level of trust rather than by other motivations [22].

Trust and cooperation have been studied across a diverse set of situations. In some situations, the trustee category may comprise specific persons (e.g., the best friend); in other situations, it may comprise more abstract categories (e.g., a stranger). For instance, Uslaner (1999) describes situations that involve trusting strangers, e.g., a fruit seller who does not attend to his stall in person but rather trusts strangers to pay by putting the correct amount into a locked mailbox [23]. While the trustees in such situations are strangers unknown to truster A, it is very likely that, in real life, individuals make certain assumptions about who will be among those strangers, what their values are, what profession they pursue, etc.

Generally, it is assumed that a truster A’s cooperative behavior in such a setting is determined by A’s expectation of B’s cooperation. In other words, it is hypothesized that *there is a positive relationship between trust and cooperative behavior*. We review empirical evidence regarding this hypothesis in Section 3. It is mixed and we provide arguments for why this is the case.

Borrowing from the idea of ideal experiments [24], we may discuss how we would ideally set up a study to investigate the link between trust and cooperative behavior. Social scientists assume that trust is linked to cooperative behavior across a wide variety of situations, e.g., trusting public officials to be sincere about their campaign promises [25], trusting a seller in a market (e.g., buying a used car) [26], trusting a political party when voting [27], trusting someone to keep one’s data secure [28], or trusting strangers to return one’s wallet [10]. Hence, we would ideally observe and measure trust and cooperation across a wide variety of such situations. In other words, we would follow a randomly sampled group of individuals through their life, and as soon as one of our individuals enters a trust situation, we would measure her trust level (ideally through accessing her thoughts unobtrusively), i.e., her expectation of whether B cooperates, and whether she actually chooses to cooperate herself. Additionally, we would collect various situational parameters, e.g., A’s and B’s background characteristics, the behavior that A expects of B, the time, the place, etc. As a consequence, we would end up with many data points both on trust and cooperative behavior allowing us to study the trust—cooperation link across a wide variety of situations. Additionally, we could design a randomized intervention in which we try to manipulate people’s trust.

This ideal scenario is unattainable, which leaves us with two strategies. A first strategy is to study *hypothetical situations*. Here, we describe situations to individuals, query them what their expectation would be in those situations and ask them whether they would cooperate or not. It is possible to do so in a standardized way across many individuals. While we can learn a lot from such an approach [26, 29], we remain in the realm of *hypothetical situations*, whose relation with reality is not entirely clear [30]. In other words, we only ask individuals how they would behave, but don’t observe whether they actually really behave in this way.

A second strategy is to try to find *real situations* in which we can measure both individuals’ trust and their cooperative behavior. Since we are unable to attach ourselves to individuals and follow them through their life, we have to identify specific situations where we are able to observe them or create such situations ourselves. A survey or a lab experiment in which we query individuals’ expectations and subsequently observe a real decision—as opposed to a hypothetical decision—belongs to this latter category. Naturally by choosing this latter approach, we can still debate on how behavior in that particular situation generalizes to behavior in other situations.

## Previous evidence and insights

The relationship between trust and cooperation has been examined across a series of studies. The setting most commonly chosen is the lab experiment that allows researchers to survey participants’ expectations and observe their behavior in a controlled environment. Interpreting corresponding evidence is challenging insofar as measures and experimental protocols vary. At the same time, slight variations thereof may strongly influence the results. Below, we review a set of influential studies in this debate and discuss some critical aspects.

Glaeser et al. (2000) was one of the first studies to investigate the trust—behavior link, triggering a series of other studies, among other things, because of its controversial result [7]. The authors measure trust as well as cooperation among Harvard students using different self-report and behavioral measures. Cooperation is measured through the amount sent in the ‘classic trust game’ [31] and the reservation value (i.e., the value that subjects place on an envelope) in an envelope drop experiment as well as through questions about past trusting behavior. Measures of (generalized) trust encompass survey questions included in the General Social Survey (GSS), the Faith-in-People Scale [32], and the Interpersonal Trust Scale [25]. Casting doubt upon research based on measures such as the most-people question, Glaeser et al. (2000, 813) find that self-report measures of past trusting behavior are better than the abstract attitudinal questions in predicting subjects’ experimental choices. These results were later supported by evidence from Brazil [33].

Fehr et al. 2002 integrate measures of both trust and cooperation in a survey among 442 Germans [8]. The authors rely on classic self-report trust measures from the General Social Survey to measure trust. Cooperation is measured through integrating a behavioral experiment (modified trust game) in the survey relying on the strategy method. We refer the reader to Fehr (2002) for an explanation of the latter. Like [7], Fehr et al. 2002 find that questions about past trusting behavior predict trusting behavior, but unlike [7], they also find that a survey measure of generalized trust predicts behavior. However, more specific measures of trust in the family, the neighborhood, the police, the courts, etc. do not predict behavior. When controlling for more concrete expectations about the amount that will be returned, the effect of generalized trust becomes insignificant. This seems in line with our argument in the previous section, namely that the effect of generalized trust runs through more concrete trusting expectations.

Gächter et al. (2004) investigate a sample of 630 participants from rural and urban Russia, students and non-students [34]. They measure cooperative behavior in a one-shot public goods game (PGG) and trust with survey questions afterwards. While they don’t find an effect for the GSS trust measure, they find an effect for the GSS trust index, a combination of three questions on contributions in the PGG. Their findings contradict [7], that measures trusting behavior in the trust game, and [35], that finds no effect of the GSS index on behavior in the prisoner’s dilemma. The effect of the GSS index seems to be due to the GSS fair and GSS help questions, once the index is decomposed [34]. Moreover, the authors find that trust in strangers has a statistically significant effect on trusting behavior as measured with a behavioral measure, while a self-report trusting behavior index shows no relationship with trusting behavior.

Bellemare and Kröger (2007) measure trusting behavior within a Dutch sample of 499 participants [36]. Trust is measured through a question querying past experiences when trusting others, cooperative behavior through an investment game. Theirs is not a direct measure of trust, rather of past experiences, and the authors find no significant effect on social capital investments, i.e., cooperation. Interestingly, however, Bellemare and Kröger (2007, 187) also query participants for their prior subjective expectations of the average amount which will be sent in the experiment as well as ask respondents to state how much they thought of receiving from senders. These expectations correlate with the decision to cooperate and invest in the game.

Ermisch et al. (2009) develop and provide rationales for a modified version of the classic trust game by Berg et al. [22, 31]. Among other things the authors argue that the binary nature of their modified game, i.e., truster as well as trustee have only two behavioral options namely keeping vs. sending money, clarifies for both truster and trustee what can be regarded as cooperative trustworthy behavior in that situation. Their modified version of the trust game is then integrated into a survey using the strategy method. This survey is fielded among persons living in the UK (N ~ 254) and generalized trust is measured with the most-people question: “Do you think that most people can be trusted or that you can’t be careful enough in dealing with people?” However, interestingly the authors also measure situational trust as a probabilistic expectation. They do so because they assume that the expectation that the trustee will do X, framed in terms of a probability is one component that leads to cooperative behavior [22]. The authors asked a subset of participants (80%) who indicated that they had weighed the chances of getting their money back after their decision: What did you think your chances of getting your money back were? While generalized trust has no effect on cooperation, they find that the person’s expectation of the chances of return is strongly related to their experimental trust decision [22].

Sapienza et al. (2013) rely on data from students of the University of Chicago (N = 502) who played a modified version of the *classic trust game* to measure cooperation which is preceded by two lotteries and an asset market game [37]. Trust is measured through querying trusters’ beliefs about the receivers’ behavior for every possible amount sent, using the strategy method in the game, after the truster took a decision. Furthermore, they ask standard questions to measure generalized trust around seven days prior to the experiment. Behavioral measures of risk aversion and other-regarding preferences are collected through two other games. The study finds that cooperative behavior in the trust game is correlated with the sender’s expectation of the receiver’s trustworthiness but also with his [risk and other-regarding] preferences [37]. Moreover, the study finds that senders’ expectations are correlated with the WVS-trust (World Values Survey) question, as well as other attitudinal questions on trust which suggests that the WVS-question captures the expectation component of the trust game [37]. In other words, it provides evidence for our suggestion in Section 2 that generalized expectations (generalized trust) tend to affect specific situational expectations (trust). Importantly, the authors also explicitly suggest that the best measure of trust as defined by Gambetta (1988) would be the expectation about the amount returned for large amounts sent, since this variable is the least contaminated by other considerations [37].

Peysakhovich et a. 2014 study the so-called cooperative phenotype, i.e., whether one can observe a domain-general tendency towards prosociality reflected in decisions in cooperation games [38]. The authors collect data on Mechanical Turk (participants living in the U.S.) and investigate to what extent behavior across different games correlates. They also collect self-report measures after the experiments and find that cooperators [in the public goods game and the dictator game] have higher generalized trust than defectors. Thereby, the authors rely on a modified version of the trust question in the World Values Survey: “How much do you agree with the statement: ‘Most people can be trusted.’?’ using a 5-point Likert Scale from ‘Completely disagree’ to ‘Completely agree’” that is asked after the experiment. Hence, as in other studies trust is measured after observing behavior in the game.

While there are several other important studies in this area [39–41], the above selection of studies is sufficient to deduce a series of insights. First, as suggested in Section 2, the ideal setup would be to study the trust—behavior link across a wide variety of real-life situations. The bulk of the literature measures cooperative behavior in very particular situations, namely economic games (often in the lab) in which individuals exchange money. Besides the role of sample characteristics [42, 43] one can also debate to what extent it is possible to generalize from cooperation in this particular setting to cooperation/behavior in other situations. Hence, finding other useful setups and situations to investigate this link seems worthwhile.

Second, evidence on the trust—behavior link is inconclusive. Since there is a strong variation in experimental protocols, measures of trust and cooperation, samples etc., it is hard to discern why this is the case. However, one strong pattern emerges: While findings differ for measures of generalized trust (e.g., the most-people question), findings are much more consistent for trust, i.e., participants’ situational expectations. We actually contend that one explanation for this mixed evidence lies in the difference between *generalized trust* and *trust* and the underlying measures used in research. Generalized trust may affect behavior; individuals with a high level of generalized trust are more likely to cooperate across a wide variety of situations. However, we would argue that this causal link exists—if at all—because generalized expectations tend to affect more specific situational expectations. In other words, generalized trust affects trust. However, the link between (situational) trust and cooperative behavior should be much stronger, as it is the situational expectation that takes a situation’s parameters into account, e.g., whether the trustee is a family member or not [26, 29]. Hence, it is not surprising that measures of situational expectations generally correlate with cooperative behavior. In other words, trust understood as situational expectation—directed at a particular trustee and a particular expected behavior—and measured as such is related to the corresponding cooperative behavior. Given the evidence above, it is harder to make the case for generalized trust. In addition, situational expectations tend to mediate more generalized expectations. Therefore we focus on the latter in our study.

Third, time matters. It should be a truster’s expectation right before she acts that determines cooperative behavior in a situation (cf. Section 2). However, in the reviewed studies, the measurement of trust sometimes occurs after the measurement of cooperation; sometimes trust is measured long before observing behavior. The decision to measure trust after behavior is defended by suggesting that eliciting beliefs affects the behavior in the experiment [36]. However, we could turn this argument around and suggest that participants rationalize their behavior post hoc and answer accordingly. If the measurement of trust occurs long before the measurement of cooperation, the measure probably does not capture the expectation associated with the actual situation.

To sum up, we assume that it is *trust* as a specific situational expectation that can (and should) be measured in the actual situation in which we observe cooperative behavior or not. Previous evidence suggests that, once we follow this advice, we are likely to uncover that trust really does matter when it comes to cooperation [7, 37]. This supports our hypothesis that *there is a positive relationship between trust and cooperative behavior*. At the same time, this connection has solely been shown with cooperative behavior in abstract experimental settings, the standard methodology in previous treatments of the subject. Importantly, a focus on and measuring trust as specific situational expectations also allows us to avoid vague questions such as the most-people question that may invite respondents to think of different situations [17, 37, 44, 45].

Below, we suggest that people’s expectations and behavior when they decide to share (or not) their data represents a situation in which we can fruitfully investigate the relationship between trust and cooperative behavior. First, this situation allows us to measure trust/cooperation for a different situation than is practice in empirical research. Second, in this situation we can measure trust right before measuring cooperation. Third, it allows us to use trust measures that are specific, e.g., specifically refer to a trustee and a behavior that is expected of the trustee. Fourth, instead of collecting individuals’ trust self-reports for a large battery of different trust measures that may influence each other, we can use a single trust survey question before observing behavior. Finally, rather than focusing on students solely we can observe other social strata in this situation as well.

## Methodology: Data, measures, and methods

**Fig 1:**
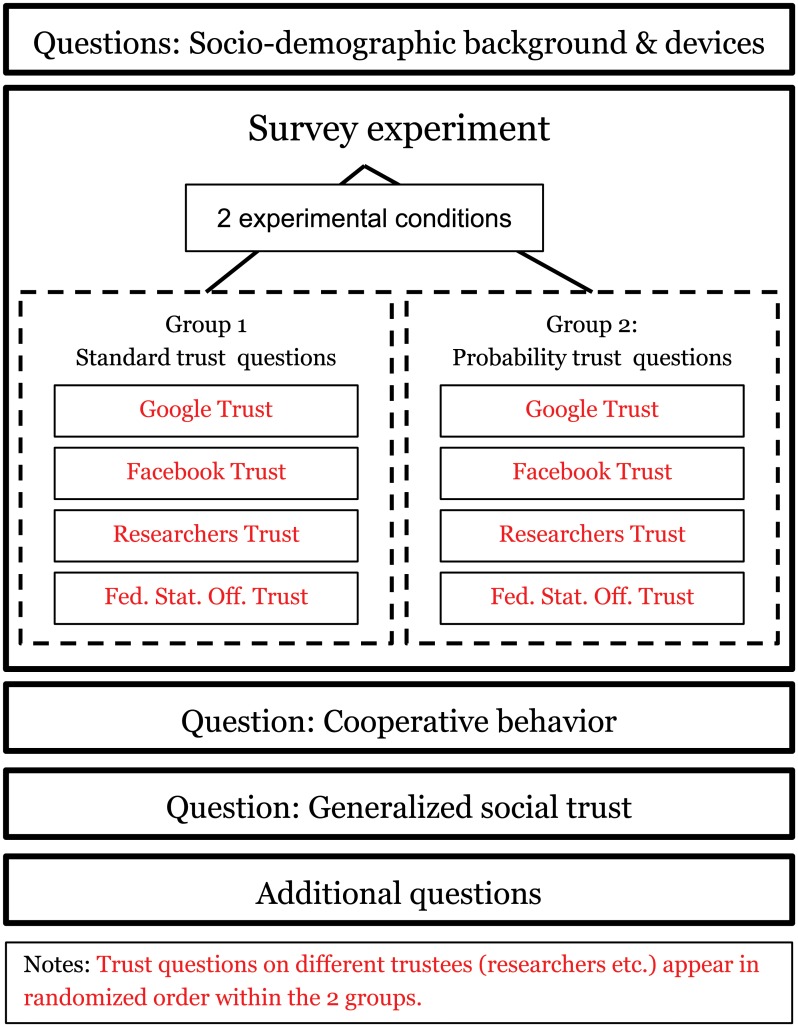
Setup of survey and experiment.

In April 2018, we conducted a web survey among members of the German non-probability online panel Mingle, operated by Respondi AG (https://www.respondi.com/). On the first page of the questionnaire, participants were informed that participation in the survey is voluntary and that the survey results would only be presented in an anonymized manner. Participants were free to skip any question of the questionnaire and break-off the survey at any point. 6088 email invitations were sent to panel members by the online panel provider. 3115 people started the survey. Quotas for gender, age, and smartphone ownership were used based on the known distribution of these characteristics in Germany; 877 panel members who started the survey were screened out because of the quotas, and 8 were screened out because they reported being under the age of 18 or not living in Germany. Out of the 2230 remaining respondents, 121 broke off the survey (5.4%). 7 respondents had duplicated IDs and their records (14) were dropped from the data set. The remaining 2095 respondents completed the online questionnaire. In our survey we measured both participants’ expectations and participants’ cooperation in terms of data sharing. We, the university researchers, were the trustee in this situation. The structure of the survey we developed is depicted in Fig 1.

Participants started with a series of questions on their socio-demographic background and what kind of electronic devices they owned. We then randomly assigned subjects to groups that received different types of trust survey questions. Some scholars define trust in terms of a subjective probability, others do not make this conceptual distinction [5, 37]. From a measurement perspective, measuring trust as a subjective probability may provide both advantages and disadvantages [17]. For instance, corresponding questions do not contain the term trust, which may carry slightly different meanings across languages. Evidence also seems to suggest that probability questions fare better in terms of predicting behavior than the standard questions used to measure generalized trust [22, 37]. Mirroring this conceptual difference, we used two different question types. This allowed us to compare the measures themselves but also to test whether one or the other was more strongly connected to our measure of cooperation.

Hence, Group 1 received *standard trust questions*, while Group 2 received *probability trust questions*, both using a numerically labeled 11-point scale with additional verbal labels at the endpoints. Standard trust questions were: “On a scale from 0 ‘not at all’ to 10 ‘fully’, how much do you trust that B will use your personal data for internal purpose only, that is, not share the data with third parties?” Probability questions were: “On a scale of 0% = ‘event will certainly not happen’ to 100% = ‘event will certainly happen’, how likely do you think it is that B uses your personal data for internal purpose only, that is, not share the data with third parties?” B, the trustee, was replaced with “Google”, “Facebook”, “university researchers”, and “the Federal Statistical Office”. In our empirical analysis, we both estimated our models by separating the data for the two different trust questions as well as by pooling the data. Moreover, in each of the groups, we queried trust in four actors—Google, Facebook, University researchers, Federal Statistics Office—in a random order to ensure that there are no order effects.

In the original survey there were four treatment arms, in two of which we collected probing data after querying the trust questions asking respondents why they picked a certain value on the trust scale (See Fig C in S1 Appendix for the randomization). As argued in Section 2, in the present study we were interested in measuring respondents’ expectation as unobtrusively as possible and did not want to artificially inflate any connection to subsequent behavior. Probing can be regarded as an intervention. It may artificially increase respondents’ mental engagement with our trust measures, which may affect subsequent responses. For instance, probing may reinforce respondents memory regarding which value they picked on the trust scale and increase their commitment to this choice. In line with this idea additional analyses seem to show that probing affects the correlation between our trust questions and our measure of cooperative behavior (available upon request). Therefore, we discarded the data from the two groups in which individuals were exposed to probing, which leaves us with a N of 1054. Tables A and B in S1 Appendix provides summary statistics on some socio-demographic variables of our sample. The average age was 44.7 and the share of women and men was equally distributed. See Table M in S1 Appendix for the survey questions used.

After our trust measures, respondents received the following question to gauge cooperative behavior (answer categories: *yes/no*):

We, the researchers at the University of Mannheim, would like to include data from the social insurance carriers for a randomly selected sample of the survey participants in our analysis. This includes, for example, additional information on previous periods of employment or unemployment. We would like to ask you to give your consent for this data to be linked to the survey data. All data protection regulations are strictly observed during the analysis, i.e., the results are always anonymous and do not allow any conclusions to be drawn about your person. Your consent is of course voluntary. You can also revoke it at any time. Should you be selected, do you agree to the data being linked?

We thus asked respondents to cooperate with us, the researchers, in terms of allowing to link their survey data to administrative records. Ultimately, respondents who agreed were informed that they were not selected. While our trust measure asks for “university researchers” more generally our measure of cooperative behavior asks participants to cooperate with researchers that come from a particular university. We contemplated using a more specific reference to Mannheim researchers in the trust question but decided that respondents are unlikely to differentiate between researchers of different universities.

Finally, we were interested in the extent to which situational trust correlates with generalized trust. We thus included the standard measure of generalized trust in the survey after our measures of situational trust and cooperative behavior: “Generally speaking, would you say that most people can be trusted, or that you can’t be too careful in dealing with people?” with a scale going from 0 “you can’t be too careful” to 10 “most people can be trusted” (cf. European Social Survey). Section 5 provides further statistics and graphs on the explanatory and outcome variables.

## Empirical results

### Normal vs. probability trust questions

While we did not formulate explicit hypotheses, our data allows us to compare normal trust questions with probability trust questions. Conceptually, the latter are more aligned with certain trust conceptions. For instance, [37] suggests that trust measured as a probabilistic expectation is best aligned with Gambetta’s classic definition of trust [5]. Both question types may have particular advantages. For instance, normal questions do not require a notion of probability that may be challenging for some respondents. Probability questions, on the other hand, avoid the potentially problematic term trust, which may be understood differently across individuals [17, 46]. We randomly assigned respondents to these two different question types.

**Fig 2:**
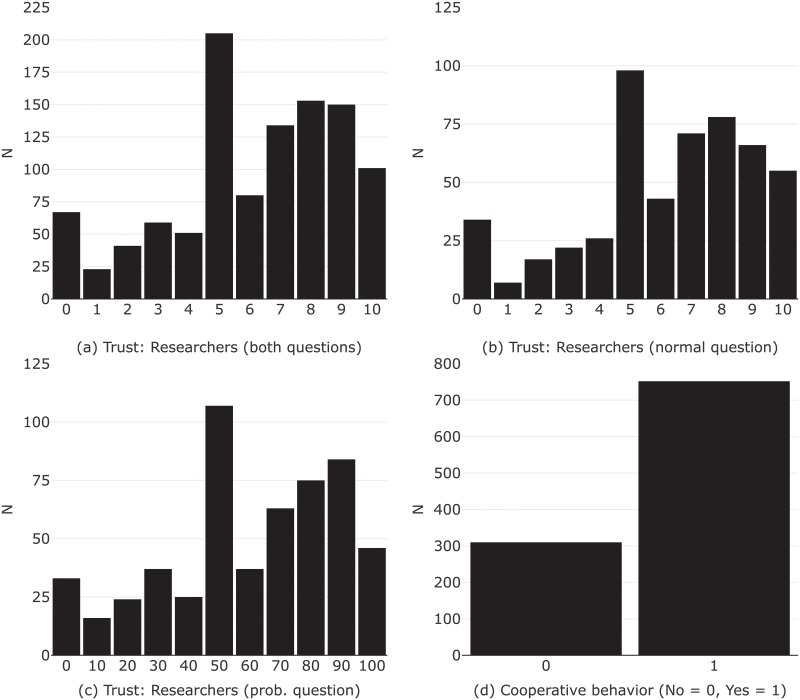
Distribution of trust and cooperation.

We find that the distributions are almost equal in terms of mean and variance (cf. Fig 2, Plot b and c). The mean of trust lies at 6.2 (Variance: 7.6) for the subsample that received the normal question (Group 1) and at 60.3 (Variance: 794.9) for the subsample that received the probability question (Group 2).

Both the standard version and a bootstrapped version of the Kolmogorov-Smirnov test, which provides correct coverage even when the distributions being compared are not entirely continuous, validate this impression [47]. The responses to both questions cluster in the middle (somewhat less on the normal trust scale), and the same is true for the generalized trust question (see Fig A in S1 Appendix).

The mean of trust lies at 6.1 for the whole sample that is combining answers to the two different questions (cf. Fig 2, Plot a). Generally, individuals in our sample cover the whole range of the trust scale. The fact that our specific trust measures do not correlate strongly with generalized trust indicates that the measures tap into a different concept (see Table E in S1 Appendix). Certainly, a much more elaborate setting is needed to test the workings and comprehension of these two questions against each other. Nonetheless, these results suggest that researchers may—aligned with their conception of trust—carefully choose one over the other without too many repercussions. Below, we’ll see that the different question types also display a similar connection to cooperative behavior.

Fig 2 also shows that a considerable share in our sample displays cooperative behavior, i.e., around 71% in our sample agree to let us link their data to more records. This attenuates any fear that the fact that our study requires participation in the first place may result in less variation in the outcome.

### The relationship between trust and cooperative behavior

To investigate the relationship between trust an cooperation, we start by estimating a series of linear probability models (LPM) [48]. Table 1 provides the results. Our outcome variable—cooperative behavior—is dichotomous, and the coefficients can be interpreted as an increase in the probability of cooperating per one-unit increase on the 11-point trust scales. We re-scaled the probability trust questions (0-100) to values from 0 to 10 to make the coefficients more comparable. Model 1 displays the results for the pooled data (both trust question types), Model 2 the result for the normal question and Model 3 for the probability question.

**Table 1:** Trust and trusting behavior.

	*Dependent variable*:					
	Cooperative behavior					
	M1	M2	M3	M4	M5	M6
Trust researchers	0.003			0.003		
	(0.01)			(0.01)		
Trust researchers (normal question)		0.01			0.01	
		(0.01)			(0.01)	
Trust researchers (prob. question 0-10)			−0.01			−0.01
			(0.01)			(0.01)
Constant	0.69***	0.62***	0.76***	0.69***	0.61***	0.75***
	(0.03)	(0.05)	(0.05)	(0.04)	(0.05)	(0.05)
Observations	1,052	513	539	848	415	433
R^2^	0.0003	0.01	0.002	0.0005	0.01	0.002

Note:

*p<0.05;

**p<0.01;

***p<0.001

Linear probability models.

The coefficients in Table 1 lie between -0.007 and 0.015; hence they are small. One could argue that our trust questions are a bit too fine-grained to see effects in our models. However, if we were to make an extreme comparison between those with a trust value of 0 and those with a trust value of 10, the difference on our cooperation measure is not large. A move from trust = 0 to trust = 10 results in an increase of the probability of data sharing by 0.03 (0.0028 × 11) taking the coefficient of Model 1, and of 0.15 (0.0139 × 11) taking the coefficient of Model 2. In our view, these differences are small given that we compared the most extreme categories. Additional models in which we add the three covariates female, age, and education lead to similar results. Standard errors and hypothesis tests are normally invalid, since LPMs’ errors violate assumptions of normality and homoskedasticity [49]. For this reason, we also provide estimations from logistic regression models in Table C in S1 Appendix. These lead us to the same conclusions. To sum up, our data does not seem to provide evidence in support of our hypothesis that there is a positive relationship between trust and cooperative behavior as measured in our study. We provide various possible explanations of why this is the case in the conclusion.

As on other scales, responses tend to cluster around the midpoint of trust scales [50, 51]. It is unclear whether this really represents a problem. It is possible, for instance, that respondents who don’t know what to answer simply pick the midpoint. Here, we refrain from such speculations but are simply interested in whether excluding midpoint answers changes any of our results. Models 4-6 have the same setup, only that we excluded respondents from the sample who picked the midpoint on our trust question(s). As we can see from Table 1, the results do not change.

### Exploring the relationship in subsamples

Above we concluded that there is no relationship between trust and cooperative behavior in our sample. However, it is possible that certain subsets of our respondents display a stronger connection between their trust levels and subsequent cooperative behavior. For instance, education has long been known to affect response behavior, e.g., seems to affect to what extent respondents differentiate between response alternatives, give don’t know answers, and erroneous answers in general [52]. Similarly, we may assume that the more highly educated reflect more on their responses. As a consequence, there could be a stronger connection between their response on the trust scale and their subsequent decision to cooperate or not. To test for the possibility of effects in subgroups we reestimate the models in Table 1 within respondent-subsets that have lower levels of education (finished secondary school or obtained a Middle school leaving certificate) and respondent-subsets with higher levels of education (finished advanced technical college entrance qualification or obtained a higher school Certificate) (For space reasons we moved the corresponding tables to S1 Appendix; see Tables K and L). However, we do not find any relevant differences. While there is less of a theoretical imperative we also re-estimated our models within other subsets as a robustness check, namely male and female respondents as well as respondents under the age of 30, between 30 and 49 years, and 50 years and older (see Table F, G, H, I and J in S1 Appendix). We do not find any relevant differences in those subgroups.

### Other factors that determine cooperative data sharing behavior

In addition to trust we collected data on a variety of other variables such as gender, age, education, usage of devices, number of social media accounts, and general privacy concern (Table M in S1 Appendix for question wording). In Table 2 we include those variables in a model together with trust to gauge whether any of them are related to cooperative behavior in terms of data-sharing. We focus on M1 in Table 2.

**Table 2:** Predictors of cooperation.

	*Dependent variable*:		
	Cooperative behavior		
	M1	M2	M3
Trust researchers	0.001		
	(0.01)		
Trust researchers (normal question)		0.01	
		(0.01)	
Trust researchers (prob. question 0-10)			−0.01
			(0.01)
Female	−0.05	−0.03	−0.06
	(0.03)	(0.04)	(0.04)
Age: 30-49 (ref.:<30)	0.01	−0.06	0.08
	(0.04)	(0.06)	(0.06)
Age: > = 50 (ref.:<30)	0.04	0.01	0.06
	(0.04)	(0.06)	(0.06)
Education: Intermed. certificate (ref. Sec. school)	0.04	−0.04	0.10
	(0.05)	(0.07)	(0.07)
Education: Adv. coll. cert. (ref. Sec. school)	−0.003	−0.09	0.07
	(0.06)	(0.09)	(0.08)
Education: High. school. cert. (ref. Sec. school)	−0.07	−0.15*	0.001
	(0.05)	(0.07)	(0.07)
Number of devices	0.02	0.02	0.02
	(0.02)	(0.02)	(0.02)
Number of accounts	0.03*	0.02	0.03
	(0.01)	(0.02)	(0.02)
General privacy concern	−0.07**	−0.07*	−0.06*
	(0.02)	(0.03)	(0.03)
Constant	0.72***	0.77***	0.68***
	(0.09)	(0.13)	(0.12)
Observations	962	466	496
R^2^	0.03	0.04	0.05

Note:

*p<0.05;

**p<0.01;

***p<0.001

Linear probability models.

While all other variables seem less relevant, general privacy concern stands out as a predictor of cooperation, i.e., whether respondents agree to letting us link their survey data to administrative records.

A move from general privacy concern = 0/Not at all concerned to general privacy concern = 3/Very concerned results in an decrease of the probability of data sharing by -0.27 (-0.27 × 4). The coefficient size is similar in models M2 and M3. General privacy concern, thus, proves to be an important predictor of data sharing. Importantly, however, we asked for privacy concern only at the end of our survey. In other words, the relationship could be inflated because respondents want to be consistent with their decision beforehand. A problem that is shared by studies that measure trust after observing cooperation in lab experiments (see above for examples).

## Conclusion and discussion

We started with the question *Is trust related to cooperation?* This question is highly relevant insofar as the importance and relevance of studying trust is often motivated through its assumed connection to cooperative behavior [4, 6, 53]. If trust self-reports are not linked to cooperative behavior, we may wonder what the purpose of studying them is in the first place [7]. Following previous scholars, we hypothesized that there is a positive relationship between trust and cooperative behavior. As outlined in Section 2 and based on previous findings reviewed in Section 3, we assumed that trust—an expectation occurring in and adapted to a particular situation—predicts cooperative behavior. At the same time, the experimental protocols underlying this evidence display certain issues, which, in turn, may affect the findings. One issue is that the cooperative behavior that scholars investigate refers to behavior in abstract experimental settings usually involving strangers which some may regard as distant to other “real” situations. For our study we identified a situation that would allow us to tackle some of the above-mentioned issues and suggested that asking people to share (more) data represents a situation in which we can measure both trust and cooperative behavior in a real setting.

We found that there is no relationship between trust and cooperation as measured in our study. A finding that is robust to the use of two different survey measures namely a normal trust question and a probability trust question. While scholars have long concluded that attitudinal measures are often only weakly correlated with behavior [54], this is not what we expected in our case.

In our view, this non-relationship may be rationalized in different ways. These ways, in turn, provide important lessons for the study of the trust—behavior nexus and open venues for future research. First, while our study mirrors the ideal setting we describe in Section 3 more closely than previous studies, there are also important differences that may explain the absence of a relationship. In the ideal setting, we would measure the expectation directly before observing a person’s (non-)cooperative behavior. In other words, the closer the measurement time points of trust t_1_ and cooperation t_2_, the stronger the link between the two probably is. We designed our study to minimize this time gap. However, it is still possible that a respondent’s trust does not play a role a few moments later when she considers cooperating. Future studies should elaborate on the meaning of time and potentially try to further decrease the time span between measuring the two concepts. Any such discussion should take into account that measurement usually represents an intervention into a participant’s mind.

Second, in contrast to several previous studies, we tried to minimize the difference between the trustee in the trust measure (“university researchers”) and in the cooperation measure (“researchers of the university X”). Previous evidence mostly linked trust in “most people”, to cooperative behavior with a “stranger” in the lab. Naturally, our setup deviates from the standard lab games that are about stranger-to-stranger interactions between two people. While we think that our study is sound in that regard, future studies could explore the effect of varying trustees on trust and cooperation. Researchers represent a particular group of trustees. Fig B in S1 Appendix illustrates that trust in other actors such as Google or Facebook is distributed very differently. Future research should acknowledge that the relationship between trust and cooperative behavior is potentially linked to who the particular trustee is in the situation and could attempt to measure and model this variation.

Third, trust is one potential determinant of cooperative behavior—given that the behavior is based on a conscious thought process. In principle, the fact that we do not find a relationship could indicate either that trust, measured as the expectation that one’s data is shared with third parties, does not play a role for the decision to cooperate in our study, i.e., to share more data, or that there are other considerations that trump any previously held expectations. The fact that individuals who are concerned about their data (e.g., reflected in low levels of trust) make no effort to protect their data actively or even give it away voluntarily is also known as the “privacy paradox” [14]. Future research should attempt to provide a more open exploration of considerations people make in their decision to cooperate across different situations. There is a large literature that explores how cooperative behavior in games relates to other concepts than trust such as altruism, lying aversion, morality, simple decision heuristics and the framing of the decision situation more general [55–60]. Potentially, decisions to share data are grounded in simple decision heuristics. Privacy concern correlates with sharing behavior in our study and potentially individuals that are concerned with their privacy follow a simple heuristic and avoid any sharing when being asked. We suggest probing respondents after having measured their cooperative behavior as to provide further insights into their decision rationales [55]. Moreover, scholars have used vignette (choice) experiments to test the impact of different situational characteristics on trust judgments and choices more generally [26, 29]. Recent research in the realm of data sharing also chooses this strategy [61]. A potentially fruitful extension would be to use vignettes (with different information) to prime individuals in order to induce differences in trust. Subsequently one can test whether the experimentally induced trust differences cause differences in a measure of cooperative behavior.

Fourth, it is possible that the absence of a link between trust and behavior is related to our sample. We argued that trust is necessary both to start a cooperative relationship and to sustain it. While there is considerable variation in our trust measure, the fact that individuals participate in our survey at all could be regarded as cooperation. It is likely that only individuals with a certain level of trust participate in surveys, experiments, etc. This selection occurs because trust itself may affect participation but also because there are other correlates that are linked to both trust and participation. Potentially, trust plays a more important role in starting a cooperative relationship than in sustaining it. We can make similar arguments for subsets of our sample. In principle, it is possible that subgroups exist in which there is a stronger trust—behavior link. In our view, those arguments require more scrutiny in future research.

Finally, as we move from the study of generalized trust to the study of situational trust and its relationship to behavior, we would ideally also reassess how these situational expectations are affected by previous positive or negative experiences. A string of studies investigates the impact of experiences on generalized trust [62–65]. However, there is less research on the impact of experiences on more refined expectations of trust, e.g., the expectations held by individuals that have to maneuver an increasingly complex world where their data is stored, traded and shared by an increasing number of actors.

## Supporting information

S1 Appendix Fig A, Distribution of generalized trust. Fig B, Distribution of trust across different trustees. Fig C, Randomization. Table A, Summary statistics: Numeric variables. Table B, Summary statistics: Categorical variables. Table C, Trust and trusting behavior. Table D, Confidence intervals. Table E, Trust and trusting behavior: Spearman correlations. Table F, Subsample—Age 30to50. Table G, Subsample—Age 50andhigher. Table H, Subsample—Age Below30. Table I, Subsample—Female No. Table J, Subsample—Female Yes. Table K, Subsample—University Degree No. Table L, Subsample—University Degree Yes. Table M, Wording of survey questions.
(PDF)
