# Supplementary material for: Trust and cooperative behavior: Evidence from the realm of data-sharing
Source: PLoS One. 2019 Aug 21;14(8):e0220115. doi: 10.1371/journal.pone.0220115 (PMC6703688; doi:10.1371/journal.pone.0220115)
Supplement: S1 Appendix — Fig A, Distribution of generalized trust. Fig B, Distribution of trust across different trustees. Fig C, Randomization. Table A, Summary statistics: Numeric variables. Table B, Summary statistics: Categorical variables. Table C, Trust and trusting behavior. Table D, Confidence intervals. Table E, Trust and trusting behavior: Spearman correlations. Table F, Subsample—Age 30to50. Table G, Subsample—Age 50andhigher. Table H, Subsample—Age Below30. Table I, Subsample—Female No. Table J, Subsample—Female Yes. Table K, Subsample—University Degree No. Table L, Subsample—University Degree Yes. Table M, Wording of survey questions. (PDF) [file pone.0220115.s001.pdf]

S1 Appendix: Trust and cooperative behavior: Evidence from the realm of data-sharing

Summary statistics

Table A and B provide summary statistics on some socio-demographic variables of our sample.

Table A: Summary statistics: Numeric variables

Statistic	N	Mean	St. Dev.	Min	Max
Female	1,054	0.49	0.50	0	1
Age	1,054	44.68	14.01	18	70
Trust researchers	1,054	6.13	2.79	0	10
Trust researchers, normal question	513	6.23	2.76	0.00	10.00
Trust researchers, prob question	541	60.31	28.19	0.00	100.00
Trust researchers , prob question 0-10	541	6.03	2.82	0.00	10.00
Cooperative behavior	1,052	0.71	0.45	0.00	1.00
Number accounts	1,030	1.97	1.30	0.00	5.00
Number devices	997	2.88	0.93	0.00	5.00

Table B: Summary statistics: Categorical variables

Variable	Distribution
**Education (Categories)**	
Advanced technical college certificate	130 (13)
Higher School Certificate	403 (39)
Intermediate school-leaving certificate	372 (36)
Secondary school	127 (12)
Missings	22/1,054 (2)
**Age (Categories)**	
30 to 49	414 (39)
50 and higher	435 (41)
Below 30	205 (19)

Logistic regression models

Standard errors and hypothesis tests of linear probability models (LPM) are normally invalid, since LPMS' errors violate assumptions of normality and homoskedasticity [1]. For this reason, we also provide estimations from logistic regression models in Table C below. None of our trust questions (in the pooled sample and subsamples that received the normal or the probability question) display a statistically significant effect on cooperative behavior. We already discussed whether those effects are practically significant, which is not the case. In Table D, we additionally provide the coefficient estimates with confidence intervals that are based on the profiled log-likelihood function for the models in Table C. In all models, the 95% intervals cover the 0.

Table C: Trust and trusting behavior

	Dependent variable:					
	Cooperative behavior					
	M7	M8	M9	M10	M11	M12
Trust researchers	0.01			0.02		
	(0.02)			(0.02)		
Trust researchers (normal question)		0.07			0.07	
		(0.03)			(0.04)	
Trust researchers (prob. question 0-10)			−0.04			−0.03
			(0.03)			(0.03)
Constant	0.81***	0.47*	1.13***	0.78***	0.44	1.09***
	(0.16)	(0.23)	(0.23)	(0.17)	(0.25)	(0.25)
Observations	1,052	513	539	848	415	433
Akaike Inf. Crit.	1,272.26	622.17	649.66	1,030.29	503.01	527.09
Note:	*p<0.05; **p<0.01; ***p<0.001					
	Logistic regression models.					

Table D: Confidence intervals

Model	2.5 %	97.5 %	Coefficient
M7	-0.034	0.061	0.013
M8	-0.002	0.134	0.066
M9	-0.104	0.031	-0.036
M10	-0.033	0.064	0.016
M11	-0.001	0.137	0.068
M12	-0.102	0.034	-0.033

Correlations

Table E provides Spearman correlations across the variables of interest. The variable ‘trust researchers’ contains the trust values of all individuals in our sample in which we pool the subsample that answered the standard question and the subsample that answered the probability question.

Table E: Trust and trusting behavior: Spearman correlations

	Trust researchers	Trust researchers (norm. question)	Trust researchers (prob. question)	Cooperative behavior	Generalized trust
Trust researchers	1 (1054)	1 (0.00; 513)	1 (0.00; 541)	0.02 (0.56; 1052)	0.28 (0.00; 1052)
Trust researchers (norm. question)	1 (0.00; 513)	1 (513)	–	0.08 (0.06; 513)	0.31 (0.00; 512)
Trust researchers (prob. question)	1 (0.00; 541)	–	1 (541)	-0.04 (0.34; 539)	0.25 (0.00; 540)
Cooperative behavior	0.02 (0.56; 1052)	0.08 (0.06; 513)	-0.04 (0.34; 539)	1 (1052)	0.15 (0.00; 1050)
Generalized trust	0.28 (0.00; 1052)	0.31 (0.00; 512)	0.25 (0.00; 540)	0.15 (0.00; 1050)	1 (1052)

Note:
P-values and number of observations in parentheses;
– = no overlap between observations for normal question and probability question;
correlations estimated on the basis of the Hmisc R package.

Trust and cooperation in sample subsets

Table F: Subsample - Age 30to50

	Dependent variable:		
	Cooperative behavior		
	M1	M2	M3
Trust researchers	0.002		
	(0.01)		
Trust researchers (normal question)		0.01	
		(0.01)	
Trust researchers (prob. question 0-10)			0.001
			(0.01)
Constant	0.70***	0.63***	0.75***
	(0.05)	(0.08)	(0.07)
Observations	413	216	197
R^2	0.0002	0.001	0.0001
Note:	*p<0.05; **p<0.01; ***p<0.001 Linear probability models. Sample subset with characteristic: Age 30to50		

Table G: Subsample - Age 50andhigher

	Dependent variable:		
	Cooperative behavior		
	M1	M2	M3
Trust researchers	0.01		
	(0.01)		
Trust researchers (normal question)		0.02	
		(0.01)	
Trust researchers (prob. question 0-10)			−0.003
			(0.01)
Constant	0.68***	0.64***	0.72***
	(0.05)	(0.07)	(0.07)
Observations	434	208	226
R^2	0.002	0.01	0.0004
Note:	*p<0.05; **p<0.01; ***p<0.001 Linear probability models. Sample subset with characteristic: Age 50andhigher		

Table H: Subsample - Age Below30

	Dependent variable:		
	Cooperative behavior		
	M1	M2	M3
Trust researchers	−0.001		
	(0.01)		
Trust researchers (normal question)		0.03	
		(0.02)	
Trust researchers (prob. question 0-10)			−0.02
			(0.02)
Constant	0.68***	0.48**	0.81***
	(0.10)	(0.15)	(0.13)
Observations	205	89	116
R^2	0.0000	0.03	0.02
Note:	*p<0.05; **p<0.01; ***p<0.001 Linear probability models. Sample subset with characteristic: Age Below30		

Table I: Subsample - Female No

	Dependent variable:		
	Cooperative behavior		
	M1	M2	M3
Trust researchers	0.01		
	(0.01)		
Trust researchers (normal question)		0.02	
		(0.01)	
Trust researchers (prob. question 0-10)			0.01
			(0.01)
Constant	0.67^{***}	0.63^{***}	0.71^{***}
	(0.05)	(0.07)	(0.07)
Observations	538	270	268
R^2	0.004	0.01	0.001
Note:	*p<0.05; **p<0.01; ***p<0.001 Linear probability models. Sample subset with characteristic: Female No		

Table J: Subsample - Female Yes

	Dependent variable:		
	Cooperative behavior		
	M1	M2	M3
Trust researchers	−0.004		
	(0.01)		
Trust researchers (normal question)		0.01	
		(0.01)	
Trust researchers (prob. question 0-10)			−0.02
			(0.01)
Constant	0.71^{***}	0.61^{***}	0.79^{***}
	(0.05)	(0.07)	(0.06)
Observations	514	243	271
R^2	0.001	0.01	0.01
Note:	*p<0.05; **p<0.01; ***p<0.001 Linear probability models. Sample subset with characteristic: Female Yes		

Table K: Subsample - University Degree No

	Dependent variable:		
	Cooperative behavior		
	M1	M2	M3
Trust researchers	0.01		
	(0.01)		
Trust researchers (normal question)		0.02*	
		(0.01)	
Trust researchers (prob. question 0-10)			−0.01
			(0.01)
Constant	0.70***	0.61***	0.79***
	(0.04)	(0.06)	(0.06)
Observations	510	253	257
R^2	0.002	0.03	0.004
Note:	*p<0.05; **p<0.01; ***p<0.001 Linear probability models. Sample subset with characteristic: University Degree No		

Table L: Subsample - University Degree Yes

	Dependent variable:		
	Cooperative behavior		
	M1	M2	M3
Trust researchers	0.002		
	(0.01)		
Trust researchers (normal question)		0.01	
		(0.01)	
Trust researchers (prob. question 0-10)			−0.001
			(0.01)
Constant	0.66***	0.61***	0.70***
	(0.06)	(0.09)	(0.07)
Observations	531	253	278
R^2	0.0002	0.002	0.0000
Note:	*p<0.05; **p<0.01; ***p<0.001 Linear probability models. Sample subset with characteristic: University Degree Yes		

Wording of survey questions

The table below outlines different coding decisions. Importantly, all coding is made transparent in the R Markdown file accompanying the study.

Table M: Wording of survey questions

Variable	Question	Coding
Trust researchers (normal question)	*On a scale from 0 "not at all" to 10 "completely", how much do you trust that university researchers only use your personal data internally, so do not share them with third parties?*	The trust questions were preceded by an intro: *The next questions deal with the topic of trust. Imagine a scale from 0 to 10. 0 means that you have no trust at all in something, 10 means that you trust something completely.*
Trust researchers (prob. question 0-10)	*On a scale of 0% to 100%, how likely do you think it is that university researchers only use your personal data internally, so does not share them with third parties?*	The trust questions were preceded by an intro: *The next questions deal with future events. Imagine a probability scale from 0% to 100%. 0% means that an event is certain not to happen, 100% means that an event is certain to happen.*; The question was subsequently recoded from values 0-100 to values of 0-10
Trust researchers	Simply combines the above two questions	For this variable respondents who answered the normal question and the probability question were joined; Beforehand the probability question was recoded to values from 0-10
Female	*Are you male or female?*	female = 1, male = 0
Age	*In which year were you born?*	2018 - year of birth
Education	*What is your highest level of school education?*	Categorical variable: Hauptschule = Secondary school; Mittlere Reife = Intermediate school-leaving certificate; Fachhochschulreife = Advanced technical college certificate; Abitur = Higher school certificate
General privacy concern	*In general, how concerned are you about your privacy?*	Categorical variable: Not at all concerned, A little concerned, Somewhat concerned, Very concerned; The variable is recoded to numeric values 0-3.
Number of devices	*Which of the following devices do you own?*	Categorical variable: Yes, No; This question was asked for Smartphone/Cell phone/Desktop computer (PC or a laptop)/Tablet/eBook reader; Subsequently, the yes answers across these device categories were summed up; If there was a missing on one of the questions the overall variable 'number of devices' was coded as missing
Number of accounts	*Do you currently have an account with a user name and password with the following services?*	Categorical variable: Yes, I currently have an account, No, I never had an account, No, not currently, but previously; This question was asked for Google (including Gmail)/Facebook/Twitter/LinkedIn/Xing; Subsequently, the yes answers across these device categories were summed up; If there was a missing on one of the questions the overall variable 'number of accounts' was coded as missing

Distribution of responses to different trust questions

Fig A visualizes the distribution of answers to the generalized trust question.

Fig A: Distribution of generalized trust

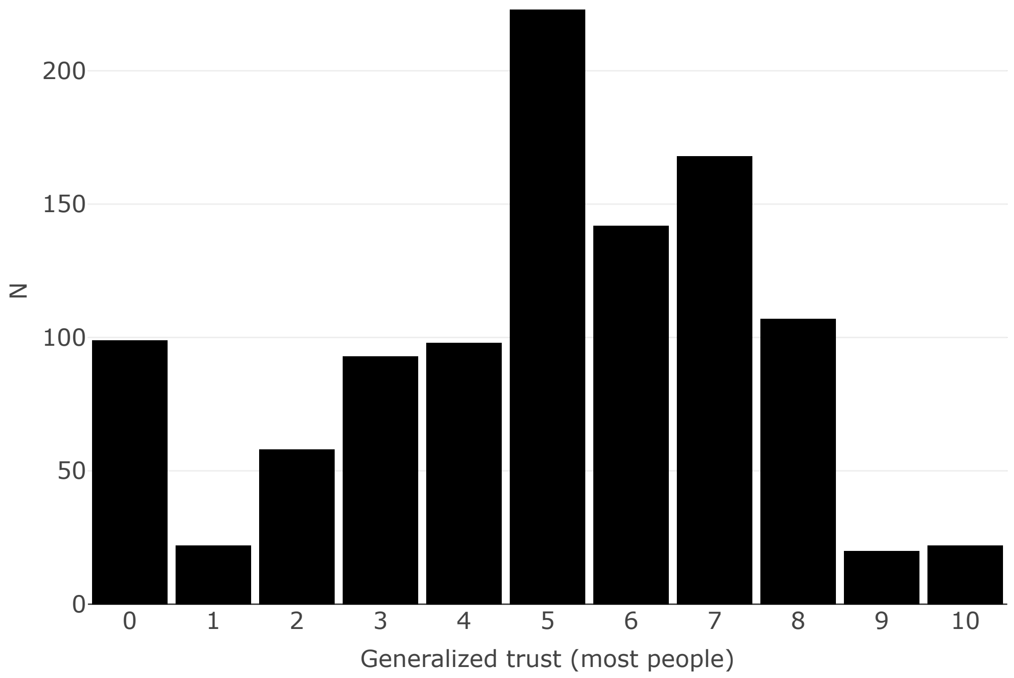

Fig B provides the distributions of trust in three other trustee categories — Google, Facebook and the Federal Statistics Office — on the normal trust question. It immediately becomes clear that trust is distributed very differently for different types of trustees.

Fig B: Distribution of trust across different trustees

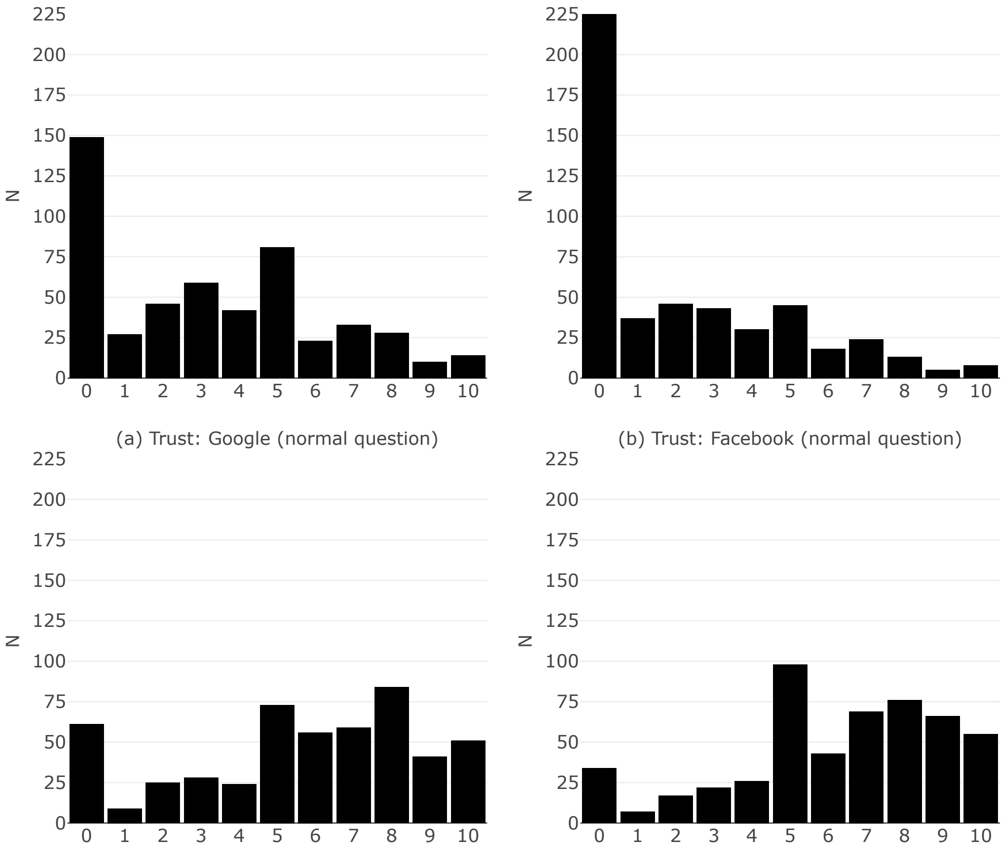

(a) Trust: Google (normal question)

(b) Trust: Facebook (normal question)

(c) Trust: Federal Statistics Office (normal question)

(d) Trust: Researchers (normal question)

Randomization

Fig C depicts the distribution across the four groups. The total N was 2095 before the data of Group 3 and 4 was discarded for our analysis.

Fig C: Randomization

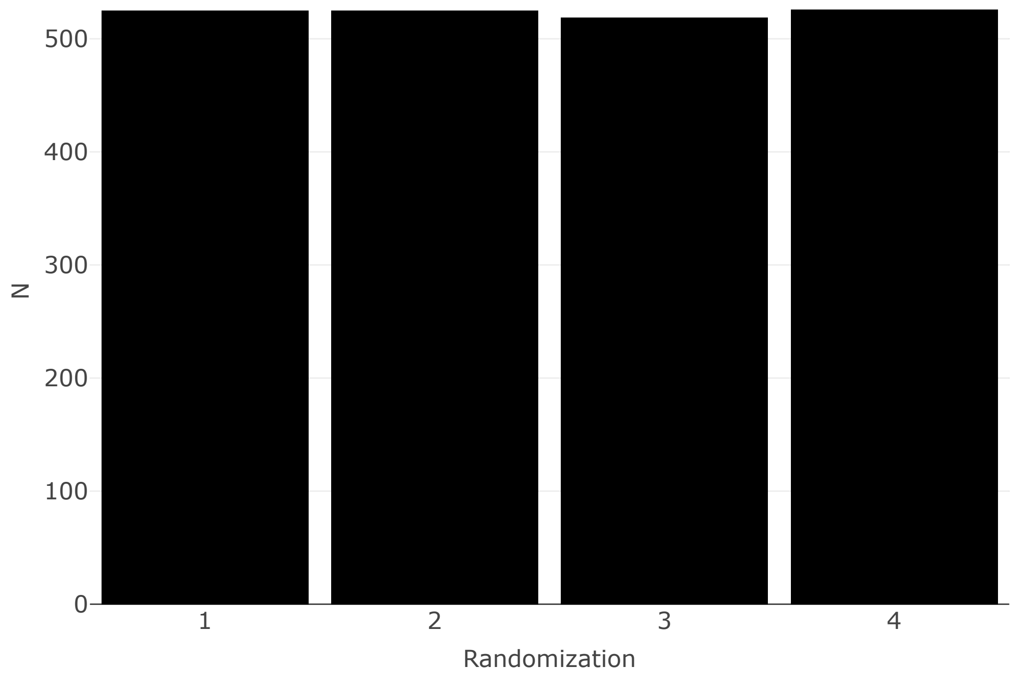

R session info

```
## R version 3.6.0 (2019-04-26)
## Platform: x86_64-w64-mingw32/x64 (64-bit)
## Running under: Windows 10 x64 (build 17763)
##
## Matrix products: default
##
## attached base packages:
## [1] stats     graphics  grDevices utils     datasets  methods   base
##
## other attached packages:
##  [1] qwraps2_0.4.1    magrittr_1.5     tidyr_0.8.3      kableExtra_1.1.0
##  [5] xtable_1.8-4     stringr_1.4.0    readr_1.3.1      stargazer_5.2.2
##  [9] dplyr_0.8.1      plotly_4.9.0     ggplot2_3.1.1    haven_2.1.0
## [13] knitr_1.22
##
## loaded via a namespace (and not attached):
##  [1] tidyselect_0.2.5  xfun_0.7          purrr_0.3.2
##  [4] colorspace_1.4-1  htmltools_0.3.6   viridisLite_0.3.0
##  [7] yaml_2.2.0        rlang_0.3.4       pillar_1.4.0
## [10] later_0.8.0       glue_1.3.1        withr_2.1.2
## [13] plyr_1.8.4        munsell_0.5.0     gtable_0.3.0
```

```
## [16] rvest_0.3.3       htmlwidgets_1.3   codetools_0.2-16
## [19] evaluate_0.13     forcats_0.4.0     ps_1.3.0
## [22] httpuv_1.5.1      crosstalk_1.0.0   Rcpp_1.0.1
## [25] scales_1.0.0      promises_1.0.1    webshot_0.5.1
## [28] jsonlite_1.6      mime_0.6          hms_0.4.2
## [31] digest_0.6.19     stringi_1.4.3     processx_3.3.1
## [34] bookdown_0.10     shiny_1.3.2       grid_3.6.0
## [37] tools_3.6.0       lazyeval_0.2.2    tibble_2.1.1
## [40] crayon_1.3.4      pkgconfig_2.0.2   data.table_1.12.2
## [43] xml2_1.2.0        assertthat_0.2.1  rmarkdown_1.12
## [46] httr_1.4.0        rstudioapi_0.10   R6_2.4.0
## [49] compiler_3.6.0
```

References

1. Scott Long J. Regression models for categorical and limited dependent variables. Thousand Oaks, London, New Delhi: Sage Publications; 1997.
